# Environmental influences on microbial community development during organic pinot noir wine production in outdoor and indoor fermentation conditions

**DOI:** 10.1016/j.heliyon.2023.e15658

**Published:** 2023-05-02

**Authors:** Aghogho Ohwofasa, Manpreet Dhami, Bin Tian, Christopher Winefield, Stephen L.W. On

**Affiliations:** a Department of Wine, Food and Molecular Biosciences, Lincoln University, Lincoln 7647, New Zealand; b Centre of Foods for Future Consumers, Lincoln University, Lincoln 7647, New Zealand; c Manaaki Whenua - Landcare Research, Lincoln, New Zealand

**Keywords:** Pinot noir, Metabarcoding, Spontaneous fermentation, Microbial diversity, *Terroir*

## Abstract

The role of microbial diversity in influencing the organoleptic properties of wine and other fermented products is well est ablished, and understanding microbial dynamics within fermentation processes can be critical for quality assurance and product innovation. This is especially true for winemakers using spontaneous fermentation techniques, where environmental factors may play an important role in consistency of product. Here, we use a metabarcoding approach to investigate the influence of two environmental systems used by an organic winemaker to produce wines; vineyard (outdoors) and winery (indoors) to the bacterial and fungal communities throughout the duration of a spontaneous fermentation of the same batch of Pinot Noir grapes. Bacterial (*R*_ANOSIM_ = 0.5814, p = 0.0001) and fungal (*R*_ANOSIM_ = 0.603, p = 0.0001) diversity differed significantly across the fermentation stages in both systems. Members of the *Hyphomicrobium* genus were found in winemaking for the first time, as a bacterial genus that can survive alcoholic fermentation. Our results also indicate that *Torulaspora delbrueckii* and *Fructobacillus* species might be sensitive to environmental systems. These results clearly reflect the substantial influence that environmental conditions exert on microbial populations at every point in the process of transforming grape juice to wine via fermentation, and offer new insights into the challenges and opportunities for wine production in an ever-changing global climate.

## 1 Introduction

The concept of *terroir* is a cornerstone of wine research globally. This term has been associated with several definitions, with all pointing to the fact that the sensory characteristics of the wine are shaped by a combination of factors such as grape microflora [1], soil and climate characteristics [2] and even the winemaking technique utilized [3].

The New Zealand wine sector has proven to be competitive in the local and international markets [4]. This may be attributed to distinctive *terroir* due to the unique environments associated with the wine making regions. These environments range from the meso-climates and diverse soils of the Marlborough wine region down to the cool climate associated with the Waitaki Valley region [4]. Additionally, a few winemakers choose a spontaneous fermentation approach for wine production, to make for an even closer relationship between their products and their *terroir* of origin.

The microbial population in a typical wine environment has mostly been studied using techniques that are cultivation-based, and these techniques can be limiting in isolating difficult to culture or rare species [5,6]. Thus, many microbes that may have important roles in wine-making processes have remained largely undetected [7]. However, the emergence of culture-independent methods such as metagenomics (untargeted amplification) and amplicon based sequencing (targeted sequencing) have proven to be useful in characterizing complex microbial communities in various fermented foods and beverages, including grape musts and various dairy products [5,8,9]. In New Zealand, relatively few studies have been done in this area. To date, most related studies only analyzed the microbiome associated with the final product [10] or across a few time points in the fermentation period [1,3,11]. One such study analyzed samples from six wine-growing regions in New Zealand and reported that across the regions examined, significant differences were found amongst the fungal communities [12]. However, winemakers from the Waipara region in New Zealand were not examined.

Pinot Noir wine sourced from the Waipara region in New Zealand is known for its unique regional characteristics [13]. This region, which is home to over 90 vineyards, has a dry, cool, and warm temperate climate and lies on the South Island of New Zealand [14]. Waipara also hosts an organic winemaker that commercially produces wines under unusual conditions; ferments are undertaken in fermentation tanks placed inside a winery, and also outside where they are exposed to the natural weather conditions of the land during the process. Zhang, Plowman [15] used MALDI-TOF MS to examine the dynamics of yeast species that were culturable during wine fermentation and demonstrated substantive differences in yeast populations between ferments undertaken indoors and out, as well as intraspecific markers in some individual species. Only one vintage was examined, and it is not known if such yeast population shifts are consistent over time, nor the possible impact on bacterial populations exposed to such variation.

In our study, using a NGS approach, we investigated the diversity of bacteria and fungi and how this changes from the beginning to the end of alcoholic fermentation, in wines produced using indigenous microflora and fermented in systems situated either indoors (winery) or outdoors (vineyard) environments. This was used to test the hypothesis that microbial populations between different fermentation systems differ as a result of the differing environmental factors they are exposed to, i.e. bacterial and fungal genera relevant to wine making would react differently in response to their immediate surroundings. We are unaware of any research that has monitored the daily microbial changes across the entire alcoholic fermentation period in two co-located environments.

## 2 Materials and methods

### 2.1 Sample collection

**Fig. 1:**
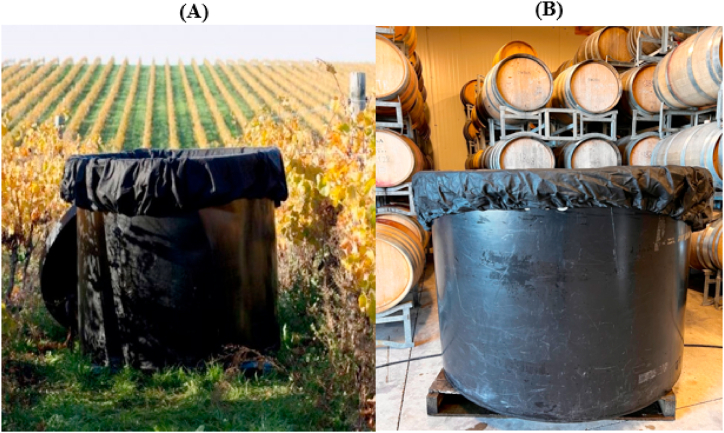
Environmental systems used in this study (A) Vineyard (outdoors) environment; (B) Winery (indoors) environmental system.

Pinot Noir grapes (21.8 °Brix) used in this study were harvested from the Greystone vineyard on the 12th of March 2021. These were then divided, and subjected to fermentation in two different environmental systems (a) In the Vineyard. This represents a natural scenery without temperature control. This is situated less than 1 KM away from the winery. (b) In the Winery. This was an indoor environment. Fermentation was done in open tanks with a volume of 1500 L (Fig. 1). From both systems, after homogenization, grape juice ferment samples were taken daily from Day 1 (at harvest) till the end of alcoholic fermentation (at pressing). Grapes used in this study were sourced from the same block of vineyard. They had 20% as whole clusters, with the remaining 80% of bunches destemmed. In both tanks, fermentation was carried out spontaneously with the same cap management systems, and besides sampling times, both were always covered with a lid to prevent insects or any sort of contaminants. For the vineyard (outdoors) fermentation, this was situated about 1 KM away from the winery (indoors). Also, a hardcover was placed on the fermenter (vineyard) if the forecast is for rain or if any precipitation starts. This way, we carefully ensured rainwater was excluded from the fermenter. During fermentation, we monitored brix levels throughout on a daily basis. On the day of pressing (the final day on skins), the wines were tested for residual sugar to ensure dryness.

After collection, samples were stored immediately at −80 °C for further analysis. To generate the pellet, the method used by Wei, Wu [10] was applied. Briefly, grape Juice stored at −80 °C was left to defrost at 4 °C. After shaking vigorously, 5 ml of each Pinot Noir grape juice ferment sample was collected into a sterile 15 ml tube and incubated at 65 °C for 1 h. Following incubation, the sample was shaken to mix thoroughly and vortexed for 60 s, and centrifuged at 3000 g for 5 min at 4 °C (Heraeus Multifuge X3R, Thermo Scientific). To the resulting pellet, 5 ml of UltraPure distilled water (Invitrogen) was added and vortexed for 60 s. Centrifugation was repeated as described previously to wash the pellet thrice, and finally, the pellet was stored at −20 °C for DNA extraction.

### 2.2 DNA extraction

DNA from all samples was extracted using a Mag-Bind Environmental DNA 96 kit (OMEGA) according to the manufacturer’s instructions, with the following modification. In order to disrupt the cells adequately, homogenization was carried out using a TissueLyser II (QIAGEN) for 3 min. The integrity of the extracted DNA was assessed using a 1.5% agarose gel electrophoresis. Total DNA concentration was determined using a DeNovix DS-11 spectrophotometer.

### 2.3 Metabarcoding

A metabarcoding approach, using a two-step amplification process as described by Ref. [16] was used in this study. In the first step, specific primer pairs were utilized in PCR amplification of the V4 region of the 16 S ribosomal RNA gene for bacteria using 515F/806R primers [16].

The large subunit (LSU) ribosomal DNA region was used for fungi analysis. An equimolar mix of the primer pairs LSU200 (A)-F/LSU481(A)-R and LSU200-F/LSU481-R were used [17]. First step PCR was done using KAPA 3G PCR plant kit and this was scaled down to a reaction volume of 15 μl. PCR conditions used were: initial denaturation at 95 °C for 120 s, followed by 35 cycles at 95 °C for 20 s (denaturation), 52.5 °C (bacterial)/55 °C (fungal) for 20 s (annealing), and 72 °C for 30 s (extension). The final extension was done at 72 °C for 10 min.

For the second PCR amplification step, the PCR products generated from the first step PCR were used as template DNA. Primers employed at this point were barcoded and the following PCR conditions were employed. Initial denaturation at 95 °C for 2 min, 5 cycles of 95 °C for 20 s (denaturation), 50 °C for 20 s (annealing), 72 °C for 30 s (extension), and final extension at 72 °C for 2 min. To normalize concentration and remove primer dimers, the resulting PCR products were purified using SeraMag Magnetic Speed-Beads [18]. DNA concentration of these libraries was then quantified using Qubit (dsDNA HS Assay Kit, Invitrogen, Carlsbad, United States). After library quantification, equimolar pooling of libraries was carried out based on amplicon length and the number of samples each library contains. DNA concentration and the quality of the final pooled library were assessed using LabChip GX Touch Nucleic Acid Analyzer (PerkinElmer, Waltham, United States). Sequencing was carried out by the Auckland Genomics Facility (University of Auckland) using Illumina MiSeq platform (phiX spike 10%, 250 × 2 cycles, NanoSeq kit).

### 2.4 Amplicon sequence variant (ASV) cluster and annotation of species

The amplicon-based DADA2 (version 1.20.0) pipeline [19] was used in analyzing the sequences obtained from Illumina MiSeq. Before using the pipeline, the downloaded sequence reads in Bcl files were converted to fastq format using bcl2fastq2 (version 2.20) Illumina software. Sequences were then demultiplexed using Claident (version 2018.05.08) [20]. DADA2 was used subsequently for quality filtering, merging, chimera removal and, inferring of ASVs. Taxonomic assignment for prokaryotes was then carried out using the SILVA v132 16 S rRNA database [21]. For eukaryotic organisms, we used the UNITE fungal taxonomic reference [22]. Where appropriate, relevant ASVs were searched using the Basic local alignment search tool (BLAST) [23] to obtain a finer taxonomic resolution. Jupyter via the New Zealand eScience Infrastructure (NeSI) HPC environment was utilized in carrying out bioinformatics analysis.

### 2.5 Statistical analysis

The open-source R programming language (v4.1.0) was used to perform statistical analysis of the microbial diversity within the winery (indoors) and vineyard samples (outdoors). The Phyloseq package [24] and several other packages (Supplementary files 1 and 2) were utilized. One fungal and bacterial phyloseq object was created. This was followed by the transformation of the ASV abundances so as to account for library size dissimilarities. Further analyses used these transformed data. To establish the core microbiome associated with each fermentation system, we recovered taxa (ASV’s) that were present in 70% of the samples with an abundance ≥0.0001.

Alpha diversity (Shannon diversity index) was calculated to determine differences within each fermentation system. This was thereafter subjected to the Shapiro-Wilk normality tests to determine suitable statistical tests to be applied. To perform a Pairwise comparison between all systems, Wilcoxon Rank sum test was used. The Betadisper in R package “vegan” was applied in estimating beta diversity and using Bray-Curtis as a distance matrix, Non-metric multidimensional scaling (NMDS) plots were made to visualize our data. The fermentation period was divided into four phases with respect to *Saccharomyces*. They are; the Lag phase (Day 1–4), Exponential phase (Day 5–11), Early stationary phase – S1 (Day 12–20), and Late stationary phase – S2 (Day 21–28 AP). To determine ASVs that were significantly different, DESeq2 was used [25]. We then tested the null hypothesis by comparing the within and between group similarities using Analysis of similarities (ANOSIM) with 999 permutations [26].

## 3 Results

### 3.1 Core bacterial community diversity and composition markedly differed across vineyard (outdoors) and winery (indoors) environmental systems

**Fig. 2:**
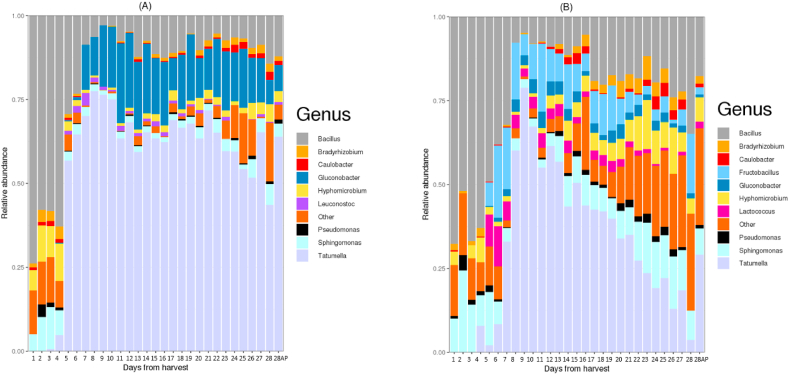
Relative abundance of bacterial genera; (A) in vineyard (outdoors) ferments; (B) winery (indoors) ferments. AP = Day 28 after press from grape skins. Bacteria with unknown genus classification are shown at the family level.

Analysis of bacterial sequence data generated revealed that relative abundance and richness changed significantly in both environmental systems as fermentation proceeded (Fig. 2).

**Fig. 3:**
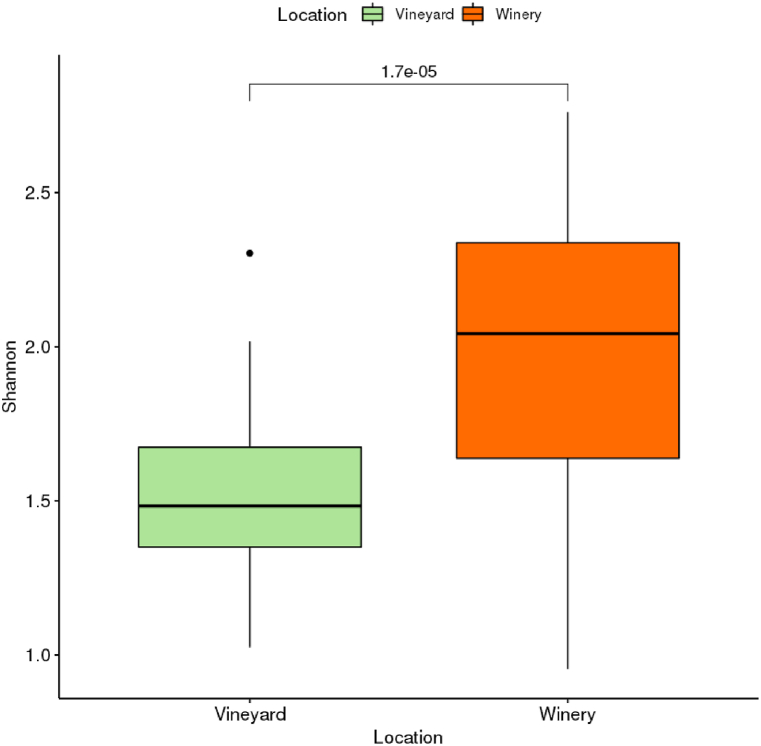
Alpha diversity (Shannon diversity index) of Vineyard (outdoors) ferments shows a lower diversity as compared to the Winery (indoors) ferments.

Alpha diversity measure (Shannon diversity index) is higher in the Winery (indoors) fermented samples (p = 1.7e-⁰⁵*). This indicates that the bacterial community associated with the winery (indoors) samples became increasingly diverse compared to the vineyard (outdoors) ferment (Fig. 3). The NMDS plot also suggested that both systems had a similar starting community.

**Fig. 4:**
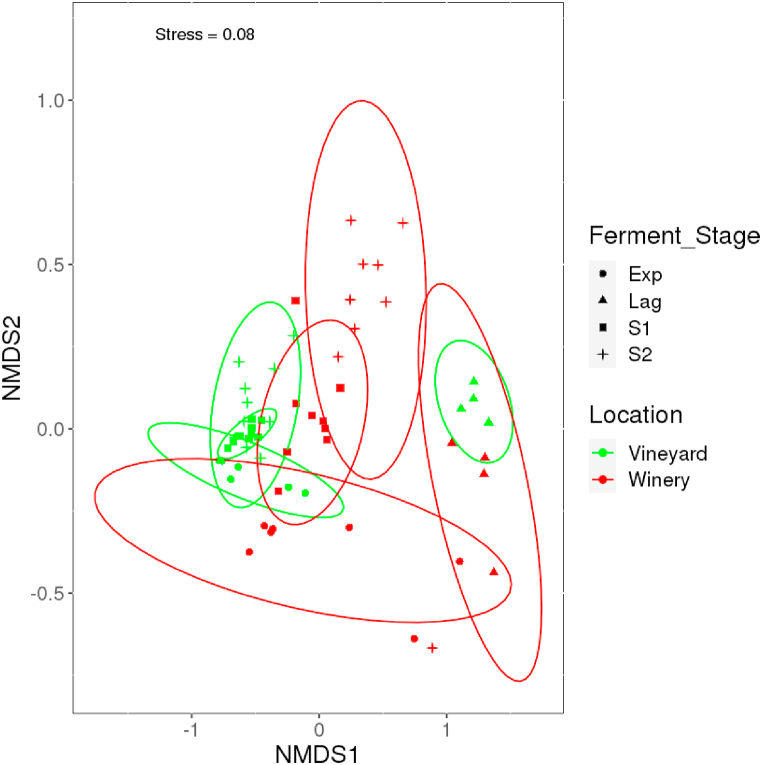
NMDS ordination for bacterial communities showing shifts in composition throughout fermentation. Samples in green and red color represent the vineyard (outdoors) and winery (indoors) environmental system respectively. Groupings that are statistically significant (*p* < 0.05) are represented with ellipses showing 95% confidence intervals. Lag = Lag phase – days 1–4; Exp = Exponential phase – days 5–11; S1 = Early stationary phase 1 – days 12–20; S2 = Late stationary phase 2 – day 21–28AP.

Observation shows that clustering was done based on environmental systems and stages. The starting community of both systems clustered together to possibly reflect the same bacterial starting community. However, as fermentation progressed, the influence of the system and ferment stage became apparent (Fig. 4).

In terms of differential abundance, analysis of the results of DESeq2 led us to conclude that 5 bacterial genera were significantly different when both systems were compared (*Tatumella, Fructobacillus, Lactococcus, Gluconobacter, Leuconostoc;* Supplementary Table 1).

#### 3.1.1 Bacterial community composition of pinot noir fermentation must

The amplicon sequence variants (ASVs) generated were used in assigning taxonomic affiliations using the SILVA database [21], which showed that both environmental systems had a total of 10 phyla. Five of the 10 phyla observed were found in musts fermented in both systems. They are; Actinobacteria*,* Bacteroidetes*,* Firmicutes*,* Planctomycetes*,* and Proteobacteria. In the Vineyard (outdoors) musts, the most dominant phyla were Proteobacteria (80.27%) and Firmicutes (18.7%).

Other phyla were present at a relatively low abundance (<1%). These include; Verrucomicrobia and*,* Chloroflexi (constituting 0.03%; Supplementary Table 2). In the Winery musts, Proteobacteria were also dominant but at 60.85% while Firmicutes was the second most abundant at 36.52%. Under-represented phyla here include; Fusobacteria*,* Deinococcus*-*Thermus*,* and Halanaerobiaeota (all making up 0.14%; Supplementary Table 2).

**Fig. 5:**
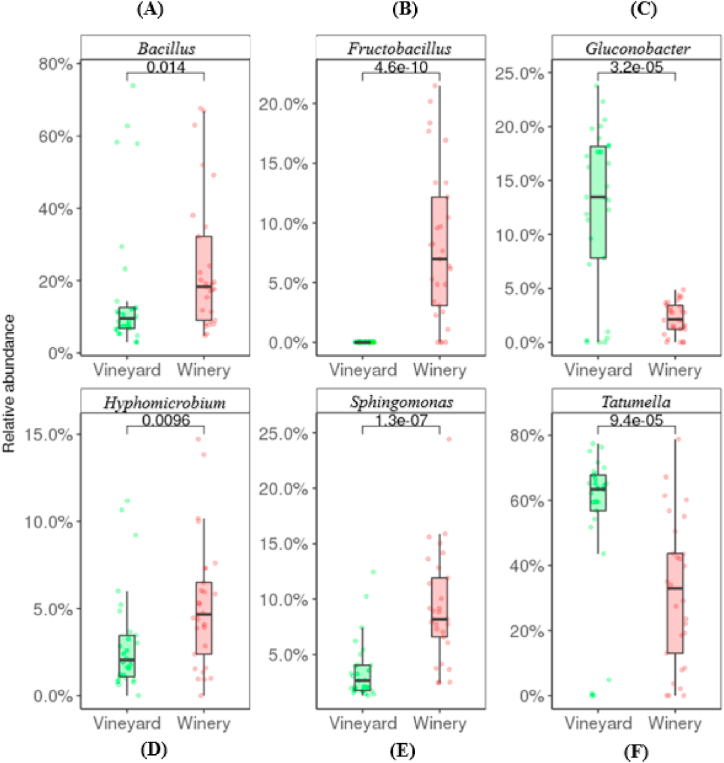
Relative abundance of (A) *Bacillus*; (B) *Fructobacillus*; (C) *Gluconobacter*; (D) *Hyphomicrobium*; (E) *Sphingomonas*; (F) *Tatumella*; across the vineyard (outdoors) and winery (indoors) environmental system.

Exploring the bacterial community in more detail resulted in a total of 164 genera. Of those, 70 genera were found in both systems while 23 and 71 genera were unique and present only in samples from the Vineyard (outdoors) and Winery (indoors) systems respectively (Supplementary Table 3). Significant differences (*R*_ANOSIM_ = 0.5814, *p* = 0.0001) were observed between both bacterial communities. Fig. 5 shows the major differences between the bacteria genera across both environmental systems.

### 3.2 Core fungal community diversity and composition were similar across vineyard (outdoors) and winery (indoors) environmental systems

**Fig. 6:**
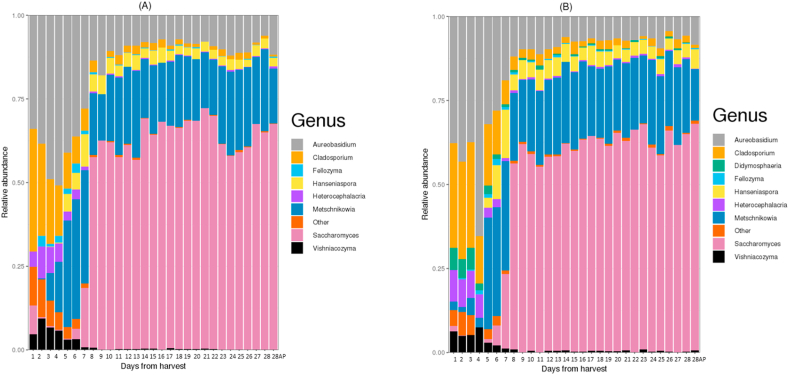
Relative abundance of fungal genera (A) in vineyard (outdoors) ferments; (B) winery (indoors) ferments. AP = Day 28 after press from grape skins.

No significant difference was observed (p = 0.24) in the overall alpha diversity of the core fungal community linked to both vineyard (outdoors) and winery (indoors) fermentation systems (Supplementary Fig. 1). Besides the lag phase (days 1–4) where few differences can be highlighted, Fig. 6 shows that the relative abundance of the core fungal microbiome was similar in both fermentation systems.

**Fig. 7:**
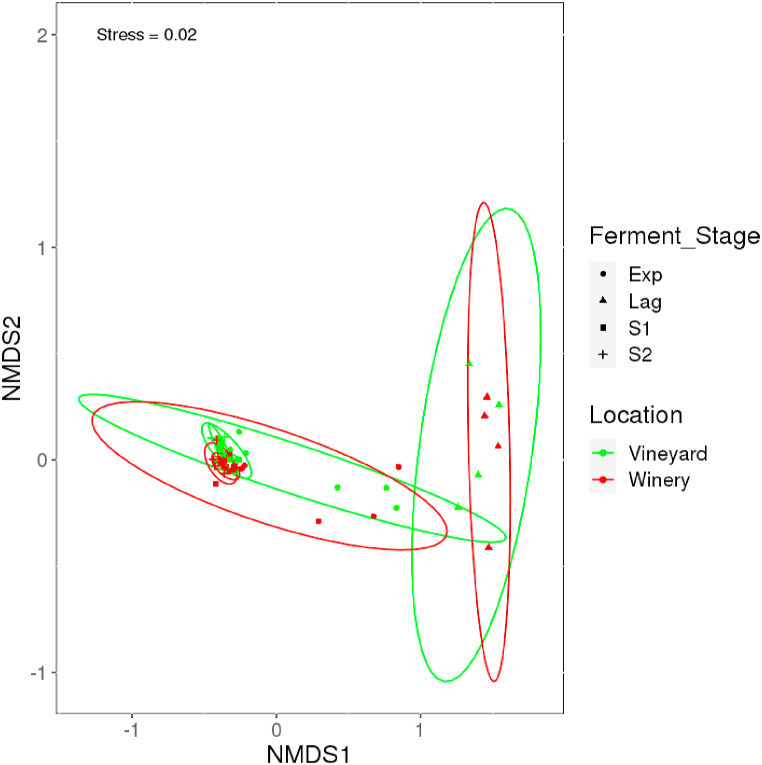
NMDS ordination for fungal communities were similar in both environmental systems. Samples in green and red color represents the vineyard (outdoors) and winery (indoors) environmental system respectively. Groupings that are statistically significant (*p* < 0.05) are represented with ellipses showing 95% confidence intervals. Lag = Lag phase – days 1–4; Exp = Exponential phase – days 5–11; S1 = Early stationary phase 1 – days 12–20; S2 = Late stationary phase 2 – day 21–28AP.

NMDS ordination also revealed the same information (Fig. 7). Segregation based on the fermentation stage reveals that the lag phase and a few samples from the exponential phase across both systems clustered together. As fermentation proceeded, no discernible differences were seen as samples in the stationary phases (S1 and S2) congregated irrespective of the environmental system. Few fungal genera made up a large portion of the core fungal microbiome due to their abundance.

We explored the compositional differences across both environmental systems, and significant differences (*R*_ANOSIM_ = 0.603, *p* = 0.0001) were observed. Most of these differences were seen only in some fermentation stages across both systems (Supplementary Figs. 2A–E).

#### 3.2.1 Fungal community composition of pinot noir fermentation must

Upon assigning taxonomy to the ASVs recovered from the fungal amplicon data, this generated 37 genera, of which 26 were found in both environmental systems. In the vineyard (outdoors), *Saccharomyces* (44.52%)*, Metschnikowia* (22.04%)*, Aureobasidium* (19.9%), *Hanseniaspora* (3.6%) and *Cladosporium* (5.4%) dominated. Few others such as *Lachancea, Candida,* and *Symmetrospora* were present only in the vineyard (outdoors) environmental system but with an abundance of less than 2% (Supplementary Table 5).

Similar trend of dominant fungi was observed in the winery (indoors) ferments. However, *Saccharomyces* made up more than half of the fungal population here (52.53%)*. Metschnikowia* was next at 21.23%, *Aureobasidium* (12.04%), *Hanseniaspora* (4.4%) and *Cladosporium* (5.17%). Other genera such as *Torulaspora, Saccharata, Fusarium, Cystobasidium, Scheffersomyces* were unique to this system but were found at low abundances. Likewise, DESeq2 did reveal that *Metschnikowia*, *Torulaspora,* and *Penicillium* were differentially expressed across both systems (Supplementary Table 4). Supplementary Fig. 4 shows the most abundant fungal genera across all systems (p > 0.05).

## 4 Discussion

Environmental conditions have been known to influence microbial diversity in different fermented food and beverages [[27], [28], [29]]. This study assessed the assembly and diversity of bacterial and fungal communities during the spontaneous fermentation of organic Pinot noir wine produced under two different environmental systems. With this, we tested the hypothesis that the microbial communities associated with wine fermentation will differ when subjected to different environmental factors. Our results indicate that varying environmental conditions seem to have more impacts on the bacterial community as compared to the fungi community. This is supported by Steenwerth, Morelan [30] who reported similar and lower fungal alpha diversity between vintages as compared to bacterial alpha diversity. Specifically, bacteria from the Enterobacteriaceae family were copious in both vineyard (outdoors) and winery (indoors), albeit differentially abundant (Supplementary Fig. 3A). This finding resembles those in prior studies by other groups [30,31]. Enterobacteriaceae spp. have previously been reported as one of the most dominant prokaryotic microorganisms associated with grapevine microbiome [32]. They are known to be beneficial as they play key roles in the microbial consortia of vineyards and grapes [32]. *Tatumella* is a well-known genus belonging to this family [1]. We quantified that Enterobacterieaceae were more abundant in the vineyard (outdoors) compared to the winery (indoors) (p.adj = 1e-^2^³). Indeed, *Tatumella* does persist all through fermentation and was the most abundant bacterial taxon – 55% in the vineyard (outdoors) and 34.2% in the winery (indoors). Such high abundance in wine fermentations has previously been observed [1,30,31,33,34]. Nisiotou, Rantsiou [35] first reported *Tatumella ptyseos* to be present in ferments using healthy and *Botrytis*-infected grapes. Having been observed in different wine fermentations around the world [[36], [37], [38]], we believe this is the first study in New Zealand to show that this genus persists throughout wine fermentation. *Tatumella* can be inhibited by the addition of SO₂ to the grape must [38,39], hence the abundance of *Tatumella* observed in our study comes as no surprise since the resultant wines were fermented spontaneously without the use of SO₂.

To establish which environmental system was more likely to support acetic acid-producing bacteria (AAB) associated with spoilage, we scrutinized the abundance of the Acetobacteraceae family and found it to be more abundant especially in the latter phase of the vineyard (outdoors) environmental system (Supplementary Fig. 3B). AAB are mostly associated with rotten grapes and are generally unwanted in winemaking [40]; genera such as *Gluconobacter* and *Acetobacter* have previously been found to produce off-flavors in resultant wine using spontaneous fermentation or “pied-de-cuve” approaches [41]. Our data further indicate that G*luconobacter cerinus* was more abundant in the vineyard (outdoors) (p.adj = 1.98e-⁰⁵) in comparison with the winery (indoors) samples. This species has been isolated from grapes in Australian vineyards [40] and wine fermentations [42]. In our case, as fermentation started with the same grapes but only differed in environmental system, this might mean that some other factors may have contributed to its differential abundance.

To ascertain if there was any differences between the environmental systems regarding the proliferation of lactic acid-producing bacteria (LAB), we compared the abundance of both Lactobacillaceae and Leuconostocaceae across the vineyard (outdoors) and winery (indoors). This was done because both families were recently synonymized and thus the Lactobacillaceae family presently refers to all genera formerly found in Lactobacillaceae and Leuconostocaceae families [43]. This family is known to undertake Malolactic Fermentation (MLF), an important process in most red wines [44]. The presence of Lactobacillaceae was not significantly different in both systems, but Leuconostocaceae was (Supplementary Fig. 3C). The genus *Leuconostoc* was detected in the vineyard (outdoors) but completely absent in the winery (indoors) (p.adj = 3.68e-⁰³)*.* On the other hand, the genus *Lactococcus* was found in the winery (indoors) but not in the vineyard (outdoors) samples (p.adj = 1.39e-^11^). Another significantly different LAB was the genus *Fructobacillus,* which appeared more abundant in the winery (indoors) than in the vineyard (outdoors) ferments (p.adj = 1.18e-^2^⁰)*.* Kordowska-Wiater, Pytka [34] have previously identified *Fructobacillus* as one of the dominant genera associated with the fermentation of Polish red wine. Its sensory contribution was underlined recently when its abundance was strongly associated with some wine volatile compounds such as ethyl isobutyrate, ethyl octanoate, and ethyl lactate [45]. A traditional LAB found in both systems was *Oenococcus*, but was only significantly different in the S2 phase of the winery (indoors) environmental systems (Supplementary Fig. 3D). Taken together, these results highlight the potential for different naturally occurring pathways to MLF between the two environmental systems. Our data imply that the genus *Leuconostoc* and/or *Oenococcus* might be responsible for MLF in the vineyard (outdoors) samples, while *Fructobacillus, Lactococcus* and/or *Oenococcus* may carry out the same in the winery samples. Nevertheless, by the abundance of the genus *Tatumella* in both environmental systems, as highlighted above, it could as well be the case that besides the traditional LAB*, Tatumella* may indeed have the capacity to carry out and complete MLF as hinted by Morgan, McCarthy [39].

*Sphingomonas and Hyphomicrobium* were also found in both the vineyard (outdoors) and winery (indoors) musts (Fig. 2 A, B). The impact of *Sphingomonas* on the organoleptic characteristics of wine is yet to be established [46]. Bokulich, Joseph [47] have previously observed that they do survive the wine fermentation process. This also supports our observation as *Sphingomonas* was present from the beginning to the end of fermentation in both systems (Fig. 2). *Hyphomicrobium* on the other hand has seldom been associated with wine and fermentation in general, although it has been detected in Japanese vineyard soils [48]. Thus, we believe ours is the first report showing its potential to persist throughout wine fermentation, though its actual role here (if any) remains to be discovered.

Though present in both the vineyard (outdoors) (15.3%) and winery (indoors) (21.5%) ferments as the second most dominant taxon, significant differences were observed in the genus *Bacillus* (Fig. 5). Members of this genus were found in abundance, especially in the first 4 days (Lag phase) of both systems, and decreased substantially thereafter, though persistent until the end at low abundances. This genus has been associated with fermented foods [49]. For sensory attributes, pyrazines which are related with vegetal and herbal aromas are said to be positively correlated with this genus [50]. In Pinot noir wine specifically, certain species of *Bacillus* have been found in wines from Western Washington State [51].

For fungi, as expected *Saccharomyces* exhibited an exponential growth trajectory (as inferred from ASVs abundance) as follows a typical fermentation [52,53]. Here, significant differences were observed only in the stationary phase 1 (S1) where it was relatively more abundant in the vineyard (outdoors) environmental system (Supplementary Fig. 2A). This might possibly be explained by alcohol concentration related to fermentation. However, no further significant differences were seen in the stationary phase 2 (S2) of both systems. This may mean that with alcohol concentration at its peak, other microbial competition was likely suppressed leaving *Saccharomyces* to thrive evenly in both systems. It should be noted that fermentation across both systems started out with the same grapes and no commercial yeasts were added. This shows that with appropriate conditions, indigenous yeasts associated with grapes can be relied upon to start and complete Pinot noir alcoholic wine fermentation as shown here.

As seen from Fig. 6 and Supplementary Fig. 2B, *Metschnikowia* was present but showed no differential abundance in both environmental systems. These non-*Saccharomyces* yeasts are well known for their role in oenology [54]. It was recently shown that *M. pulcherrima* had the potential to produce lower alcohol wines when co-inoculated with *S. cerevisiae* [55]. Interestingly, both *Saccharomyces* and *Metschnikowia* coexisted till the end of fermentation in both systems (Fig. 6 A, B). This concurs with Vicente, Ruiz [56], who highlighted that *Metschnikowia* abundance pattern in grapes and grape musts are like those of *Saccharomyces.* In terms of differential abundance, two species were found to be dominant in the vineyard (outdoors) while one species was numerous in the winery (indoors). However, we could not resolve these with certainty beyond the genus level (Supplementary Table 4).

Other variations were revealed at the genus level. For instance, the genus *Torulaspora* was only found in the winery (indoors) and not in the vineyard (outdoors) fermentation musts (Supplementary Fig. 2C). Investigating the relevant ASV using NCBI BLAST indicated that the species was *T. delbrueckii*. This species is a prominent non-*Saccharomyces* yeast as a result of its resistance to ethanol [57,58]. In co-fermentation with *S. cerevisiae* [59] or *Oenococcus oeni* as starters [60], *T. delbrueckii* has been reported to improve red wine quality. Explicitly, sensory improvements such as fruitiness [61] and mouthfeel characteristics [62] have been associated with *T. delbrueckii.* For our observation, this might indicate that the winery (indoors) environmental system favors the presence of *Torulaspora* as against the vineyard (outdoors) system. Therefore, differences in sensory attributes could be expected between the wines made from both the winery (indoors) and vineyard (outdoors) ferments in this study; we are currently exploring this hypothesis.

The genus *Hanseniaspora* was copiously present in both the vineyard (outdoors) and winery (indoors) environmental systems. Along with *Saccharomyces and Metschnikowia*, it was consistently detectable until the end of fermentation. Significant abundant differences are shown in stationary phases 1 and 2 (S1 and S2) where its relative abundance was higher in the winery (indoors) as compared to the vineyard (outdoors) environmental system (Supplementary Fig. 2E). This genus has been reported to be advantageous for winemaking purposes [63]. Specifically, some species are associated with the honey, fruity and flowery aroma of wines by way of improving the production of 2-phenylethyl acetate [64].

*Aureobasidium* was also found in both systems and showed a higher significant abundance in the S2 phase of the vineyard (outdoors) system (Supplementary Fig. 2F). Several research groups have described them to be abundant in the early stages of fermentation [65,66]. Here, it remains to be established if it does thrive well under the high ethanol concentration found in a typical fermentation environment. It is also possible that the high abundance of *Aureobasidium* detected in the end of fermentation might be as a result of the residual DNA coming from the initial population. More studies will be needed to confirm this. Further scrutiny gave a direct hit of *A. pullulans.* This yeast-like fungus has been associated with the production of several antimicrobial compounds [67]. Furthermore, its sensory attributes in wine and the vinification process have been discussed [68,69]*.* Going by the aforementioned and other properties of *A. pullulans* in other research fields, this could be a key player in wine production [66].

Our results confirm the impact of fermentation on microbial community dynamics, which appears greater on the bacterial community than the fungal community, most likely a result of their overall relative sensitivity to acid and ethanol-containing environments. Additionally, more diversity was observed in the bacterial community than in the fungal community in the early stages of the fermentative process. Taking a cue from the “winery effect” [70] where *S. cerevisiae* and other yeast species dominate the cellar and carry out alcoholic fermentation, it appears that certain bacterial and fungal organisms were better suited to the different environmental conditions of the vineyard (outdoors) and winery (indoors). This primarily explains the microbial community differences we observed here. Microclimatic factors such as temperature variation, and differential exposure to microbial dispersal by wind, and access of environmental microbes are the likely contributors of the different environmental factors across the two environmental systems.

In a 2018 study carried out in the same Waipara-based winery where cultured yeast strains were characterized by MALDI-TOF, major differences in the species- and intraspecific identities of certain yeast species between the two systems were observed [15], thus correlating with our results. Interestingly, several non-*Saccharomyces* yeasts such as *Starmerella bacillaris* and *Pichia kluyveri* which were reported in that study were completely absent from ours. This points to the possibility that microbial diversity might differ from year to year, potentially due to climatic conditions. Further studies would be required to explore this hypothesis.

## 5 Conclusions

We believe our study to be the first to monitor both bacterial and fungal populations in the winemaking process across the entire alcoholic fermentation period using a daily sampling approach. We show that the bacteria genera *Tatumella* and *Hyphomicrobium* can possibly survive alcoholic fermentation. It also indicates that *T. delbrueckii* and *Fructobacillus* might be sensitive to environmental conditions. *Tatumella* was able to thrive throughout fermentation and in both environment. Hence, more research will be required to establish the role and characteristics of *Tatumella* in a typical wine fermentation environment. Overall, given the observations in microbial populations in the different fermentation systems, we believe our study offers an interesting perspective on the “*terroir*” concept. We estimate that sensory analyses would be invaluable to determine the sensory characteristics of the resulting wines from each environmental system, to correlate unique sensory properties with the differential microbial abundance information. We do acknowledge that using metabarcoding to detect the presence of microorganisms does not necessarily mean that they are active. Thus, in the future, we look to compliment this study with culture-based approaches.

Finally, our results indicate that the effects of climate and environmental changes on microbial populations need to be better understood as this would shape future food management and production practices. We believe this is a step in that direction for New Zealand, and even global, wine industry.

## Author contribution statement

Aghogho Ohwofasa: Performed the experiments; Analyzed and interpreted the data; Wrote the paper.

Manpreet Dhami: Analyzed and interpreted the data; Contributed analysis tools; Wrote the paper.

Bin Tian: Conceived and designed the experiments; Contributed materials; Wrote the paper.

Christopher Winefield: Conceived and designed the experiments; Contributed materials, analysis tools; Wrote the paper.

Stephen L. W. On: Conceived and designed the experiments; Contributed materials; Wrote the paper.

## Data availability statement

Data associated with this study has been deposited at Sequencing data are available on the NCBI Sequence Read Archive (SRA) (PRJNA869908). DADA2 workflow used and other supplementary figures and tables are accessible at: https://github.com/Ohwofasa1/Pinot_noir_Wine_Data_Analysis.

## Additional information

No additional information is available for this paper.

## Declaration of competing interest

The authors declare that they have no known competing financial interests or personal relationships that could have appeared to influence the work reported in this paper.

## Appendix A Supplementary data

Multimedia component 9 Multimedia component 9

figs1 figs1

figs2 figs2
