# Supplementary material for: Environmental influences on microbial community development during organic pinot noir wine production in outdoor and indoor fermentation conditions
Source: Heliyon. 2023 May 2;9(5):e15658. doi: 10.1016/j.heliyon.2023.e15658 (PMC10189187; doi:10.1016/j.heliyon.2023.e15658)
Supplement: Multimedia component 2 [file mmc2.pptx]

## Slide 1
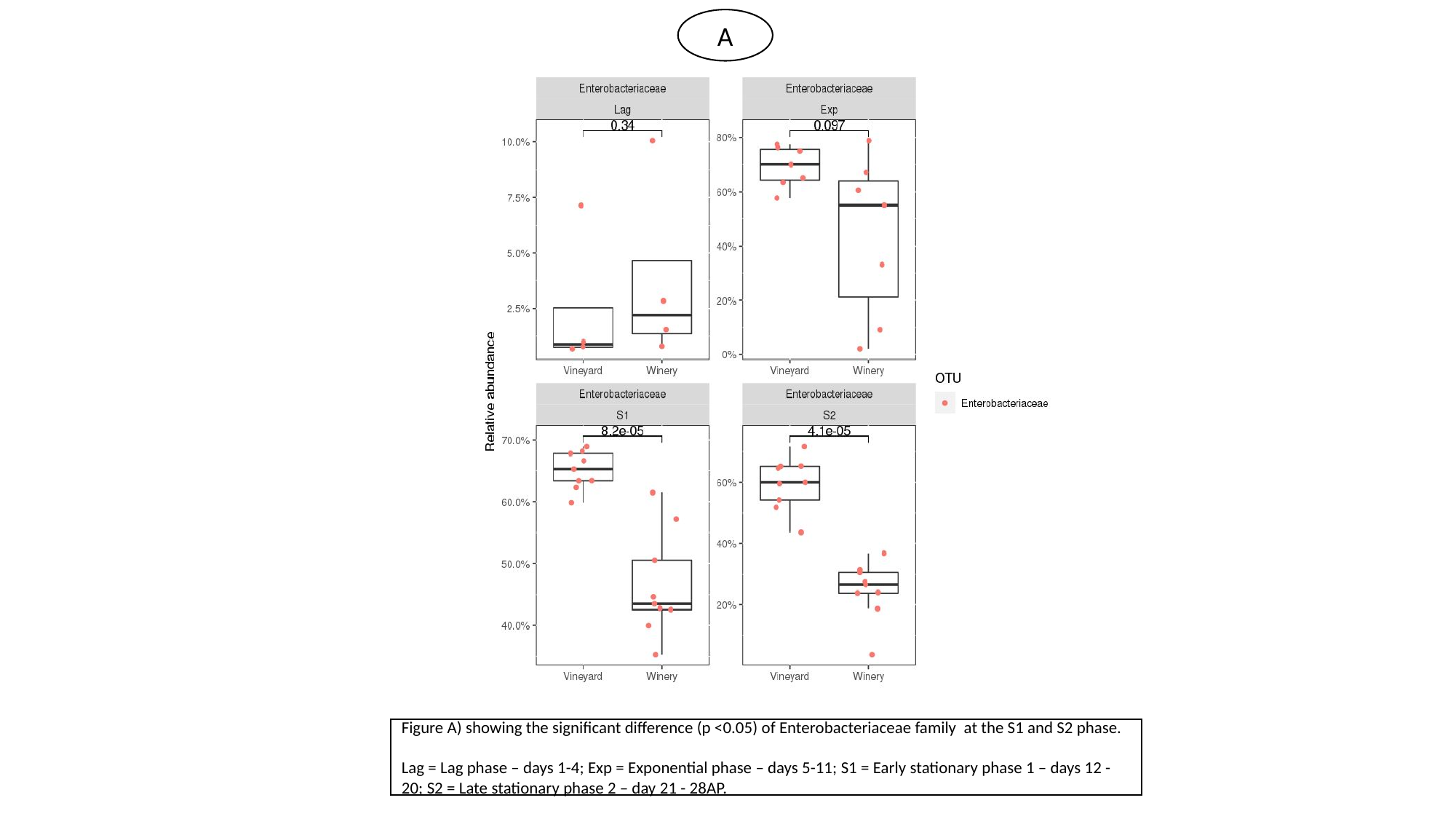

A
Figure A) showing the significant difference (p <0.05) of Enterobacteriaceae family at the S1 and S2 phase.
Lag = Lag phase – days 1-4; Exp = Exponential phase – days 5-11; S1 = Early stationary phase 1 – days 12 - 20; S2 = Late stationary phase 2 – day 21 - 28AP.

## Slide 2
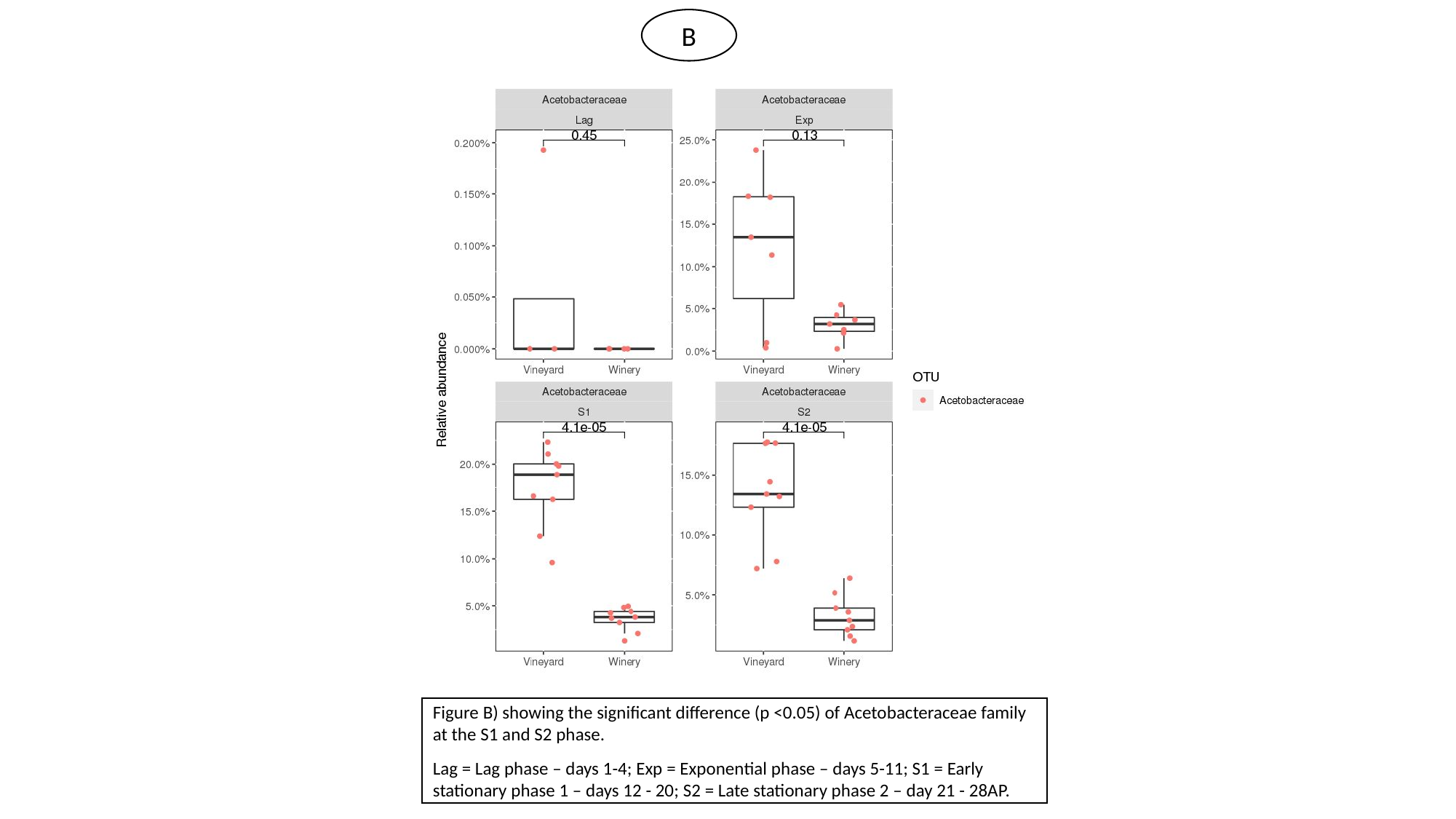

B
Figure B) showing the significant difference (p <0.05) of Acetobacteraceae family at the S1 and S2 phase.
Lag = Lag phase – days 1-4; Exp = Exponential phase – days 5-11; S1 = Early stationary phase 1 – days 12 - 20; S2 = Late stationary phase 2 – day 21 - 28AP.

## Slide 3
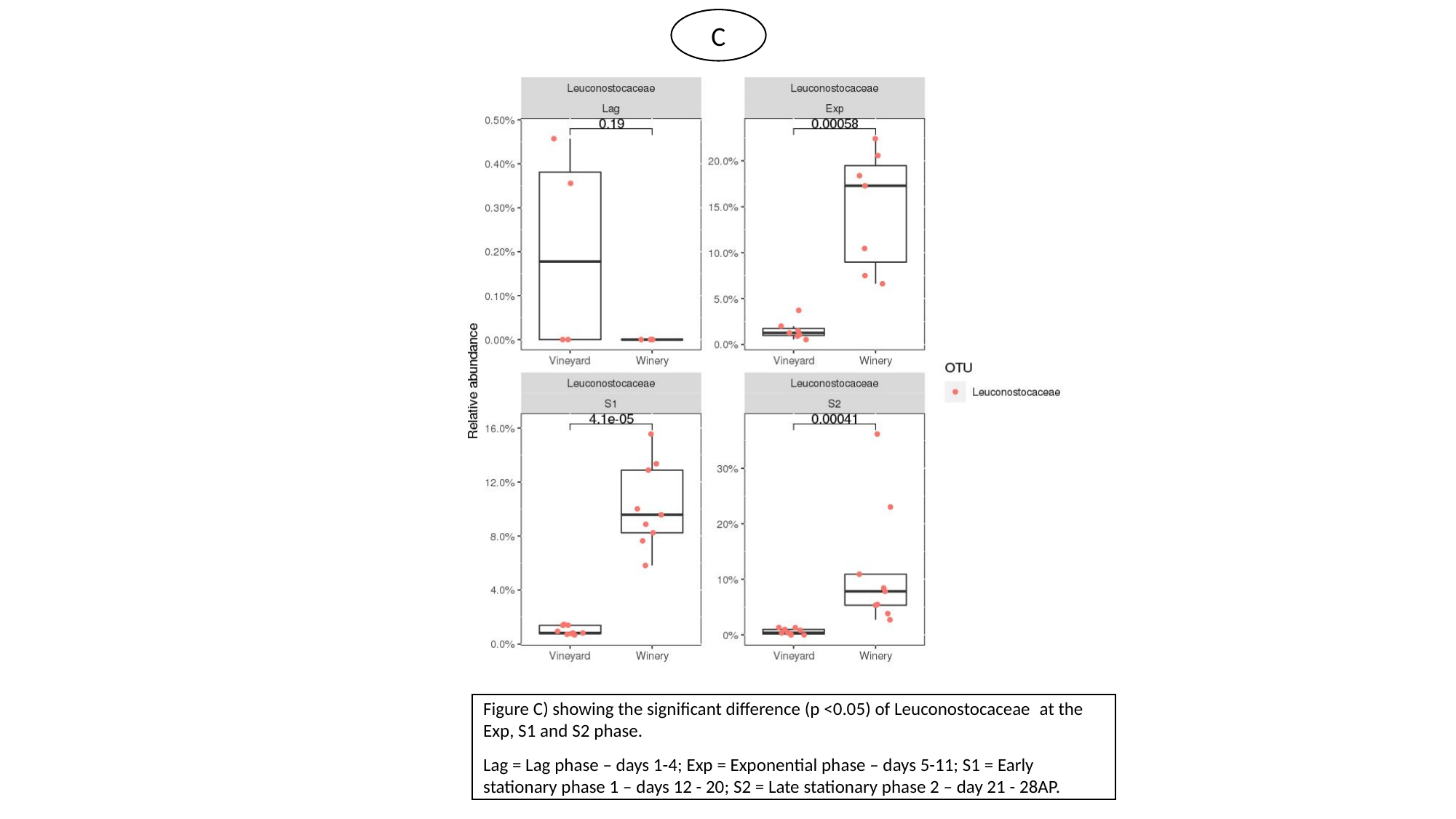

C
Figure C) showing the significant difference (p <0.05) of Leuconostocaceae at the Exp, S1 and S2 phase.
Lag = Lag phase – days 1-4; Exp = Exponential phase – days 5-11; S1 = Early stationary phase 1 – days 12 - 20; S2 = Late stationary phase 2 – day 21 - 28AP.

## Slide 4
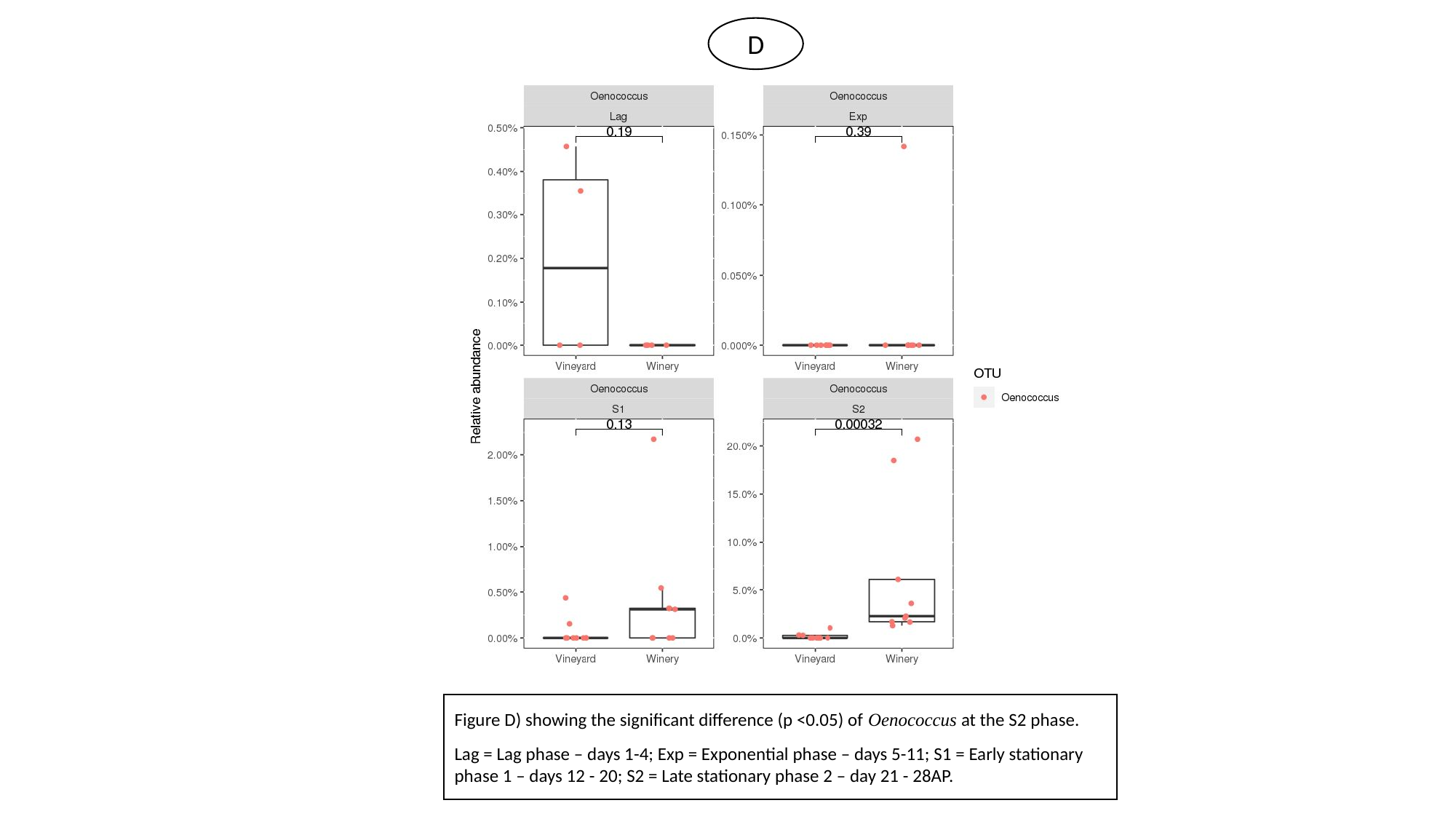

D
Figure D) showing the significant difference (p <0.05) of Oenococcus at the S2 phase.
Lag = Lag phase – days 1-4; Exp = Exponential phase – days 5-11; S1 = Early stationary phase 1 – days 12 - 20; S2 = Late stationary phase 2 – day 21 - 28AP.
