# Supplementary material for: Environmental influences on microbial community development during organic pinot noir wine production in outdoor and indoor fermentation conditions
Source: Heliyon. 2023 May 2;9(5):e15658. doi: 10.1016/j.heliyon.2023.e15658 (PMC10189187; doi:10.1016/j.heliyon.2023.e15658)
Supplement: Multimedia component 9 [file mmc9.pdf]

Supplementary Table 7: Sample Data overview.

Sample ID	Environmental System (Location)	Days from Harvest	Sample ID	Environmental System (Location)	Days from Harvest
**OPF1D1**	Vineyard (Outdoors)	1	**OPF7D1**	Winery (Indoors)	1
**OPF1D2**	Vineyard (Outdoors)	2	**OPF7D2**	Winery (Indoors)	2
**OPF1D3**	Vineyard (Outdoors)	3	**OPF7D3**	Winery (Indoors)	3
**OPF1D4**	Vineyard (Outdoors)	4	**OPF7D4**	Winery (Indoors)	4
**OPF1D5**	Vineyard (Outdoors)	5	**OPF7D5**	Winery (Indoors)	5
**OPF1D6**	Vineyard (Outdoors)	6	**OPF7D6**	Winery (Indoors)	6
**OPF1D7**	Vineyard (Outdoors)	7	**OPF7D7**	Winery (Indoors)	7
**OPF1D8**	Vineyard (Outdoors)	8	**OPF7D8**	Winery (Indoors)	8
**OPF1D9**	Vineyard (Outdoors)	9	**OPF7D9**	Winery (Indoors)	9
**OPF1D10**	Vineyard (Outdoors)	10	**OPF7D10**	Winery (Indoors)	10
**OPF1D11**	Vineyard (Outdoors)	11	**OPF7D11**	Winery (Indoors)	11
**OPF1D12**	Vineyard (Outdoors)	12	**OPF7D12**	Winery (Indoors)	12
**OPF1D13**	Vineyard (Outdoors)	13	**OPF7D13**	Winery (Indoors)	13
**OPF1D14**	Vineyard (Outdoors)	14	**OPF7D14**	Winery (Indoors)	14
**OPF1D15**	Vineyard (Outdoors)	15	**OPF7D15**	Winery (Indoors)	15
**OPF1D16**	Vineyard (Outdoors)	16	**OPF7D16**	Winery (Indoors)	16
**OPF1D17**	Vineyard (Outdoors)	17	**OPF7D17**	Winery (Indoors)	17
**OPF1D18**	Vineyard (Outdoors)	18	**OPF7D18**	Winery (Indoors)	18
**OPF1D19**	Vineyard (Outdoors)	19	**OPF7D19**	Winery (Indoors)	19
**OPF1D20**	Vineyard (Outdoors)	20	**OPF7D20**	Winery (Indoors)	20
**OPF1D21**	Vineyard (Outdoors)	21	**OPF7D21**	Winery (Indoors)	21

**OPF1D22**	Vineyard (Outdoors)	22	**OPF7D22**	Winery (Indoors)	22
**OPF1D23**	Vineyard (Outdoors)	23	**OPF7D23**	Winery (Indoors)	23
**OPF1D24**	Vineyard (Outdoors)	24	**OPF7D24**	Winery (Indoors)	24
**OPF1D25**	Vineyard (Outdoors)	25	**OPF7D25**	Winery (Indoors)	25
**OPF1D26**	Vineyard (Outdoors)	26	**OPF7D26**	Winery (Indoors)	26
**OPF1D27**	Vineyard (Outdoors)	27	**OPF7D27**	Winery (Indoors)	27
**OPF1D28**	Vineyard (Outdoors)	28	**OPF7D28**	Winery (Indoors)	28
**OPF1D28AP***	Vineyard (Outdoors)	28	**OPF7D28AP***	Winery (Indoors)	28

D = day; **AP*** = Day 28 after press from grape skins
