# Supplementary figures and images for: Environmental influences on microbial community development during organic pinot noir wine production in outdoor and indoor fermentation conditions
Source: Heliyon. 2023 May 2;9(5):e15658. doi: 10.1016/j.heliyon.2023.e15658 (PMC10189187; doi:10.1016/j.heliyon.2023.e15658)

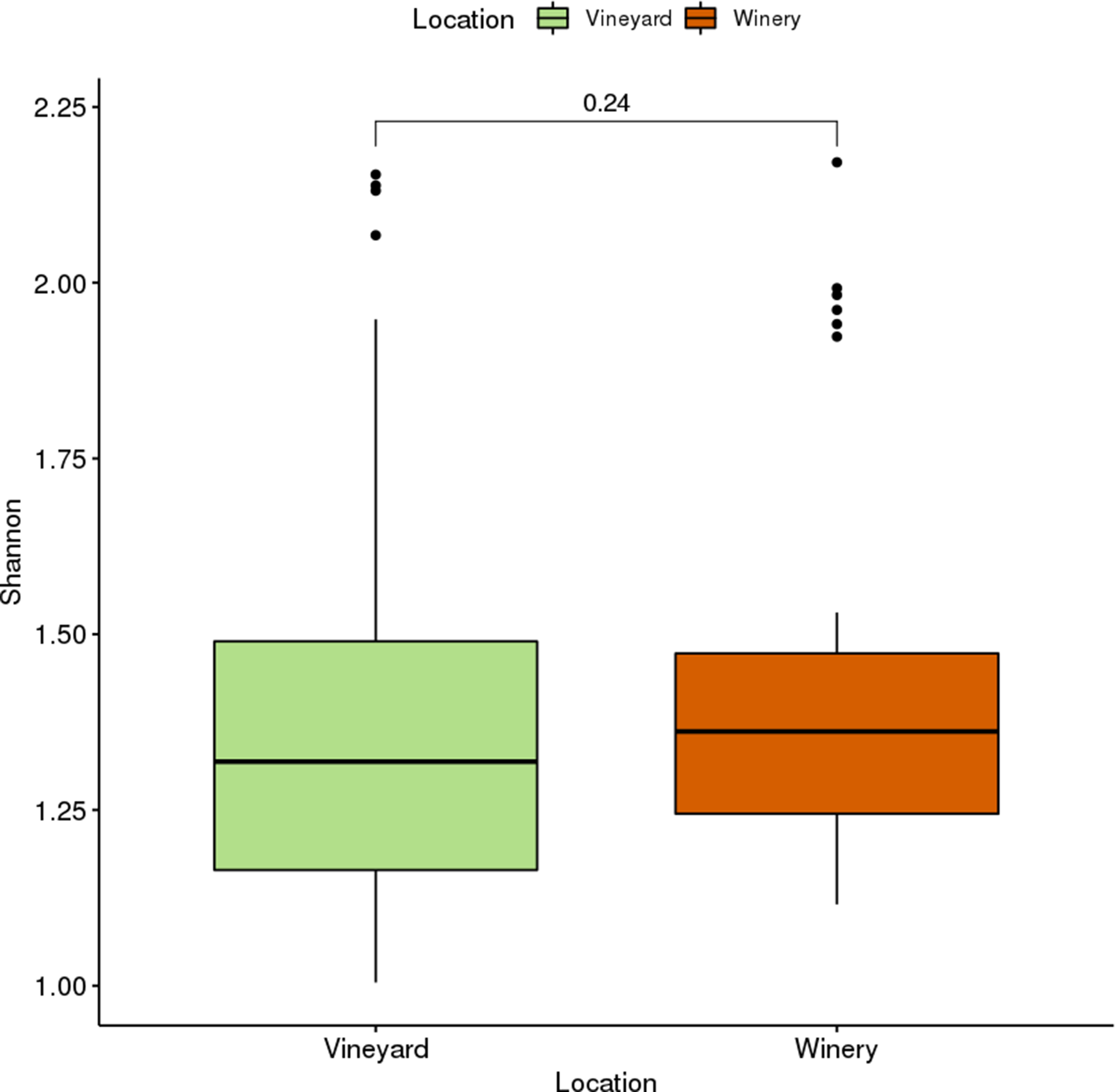

Supplement: figs1 [file mmcfigs1.jpg]

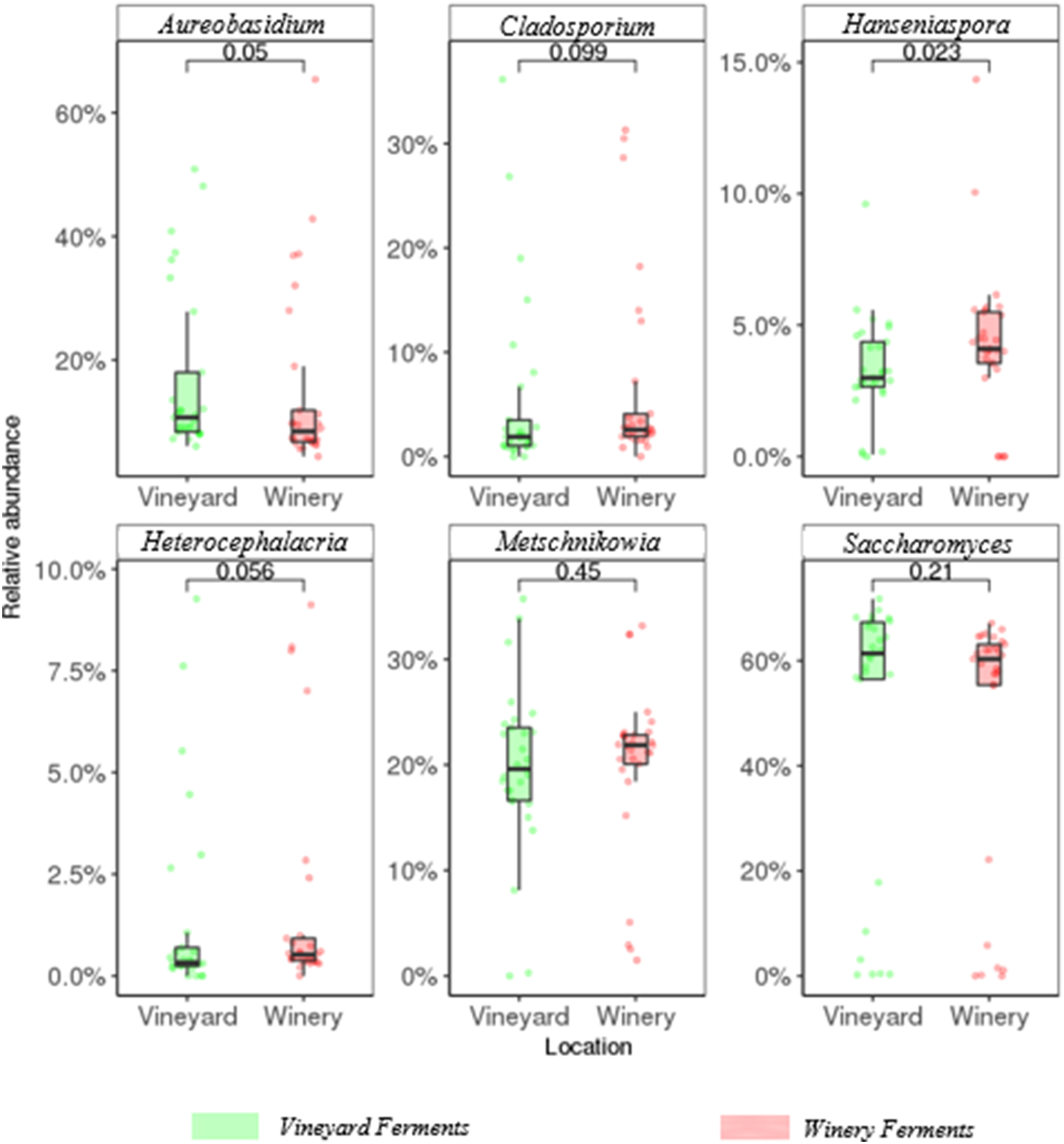

Supplement: figs2 [file mmcfigs2.jpg]
